# Efficacy of praziquantel against urinary schistosomiasis and reinfection in Senegalese school children where there is a single well-defined transmission period

**DOI:** 10.1186/s13071-015-0980-5

**Published:** 2015-07-10

**Authors:** Bruno Senghor, Omar Talla Diaw, Souleymane Doucoure, Seydou Nourou Sylla, Mouhamadane Seye, Idrissa Talla, Cheikh Tidiane Bâ, Adiouma Diallo, Cheikh Sokhna

**Affiliations:** Institut de Recherche pour le Développement, UMR 198 (URMITE), Campus International de Hann, IRD, BP 1386, Dakar, CP 18524 Sénégal; Département de Biologie Animale, Université Cheikh Anta Diop de Dakar, Dakar, BP 5005 Senegal; UFR Sciences Appliquées et Technologies, Université Gaston Berger de Saint Louis, Saint Louis, BP 234 Senegal; Institut Sénégalais de Recherches Agricoles, ISRA, route des Hydrocarbures, Bel Air, Dakar, Senegal; Programme national de lutte contre les bilharzioses et les géo-helminthiases, ministère de la santé et de l’action sociale, Dakar, Sénégal

**Keywords:** Urinary schistosomiasis, Seasonal transmission, Praziquantel treatment, Reinfection, Niakhar, Senegal

## Abstract

**Background:**

Human schistosomiasis is a significant health problem in Sub-Saharan Africa. In Niakhar, West central Senegal, the transmission of *S. haematobium* occurs seasonally between July and November. No control measures have been implemented despite high prevalence reported in previous studies. This aim of this study was to i) determine the current prevalence of *S. haematobium* in children at Niakhar, ii) assess the efficacy of one dose of PZQ (40 mg/kg) against *S. haematobium* and iii) monitor reinfection.

**Methods:**

The current study was carried out in a cohort of 329 children aged five to 15 years enrolled from six villages in Niakhar to determine the efficacy of one dose of PZQ, as well as reinfection. Parasitological screening was performed in June 2011 to determine the baseline prevalence of *S. haematobium*, and then a single dose of PZQ was administered to all selected subjects in the transmission season in August 2011. The efficacy of PZQ treatment and reinfection were monitored respectively five weeks after in September 2011 and from February to March 2012.

**Results:**

At baseline, the overall prevalence and the heavy intensity of infection were 73.2 % and 356.1eggs/10 ml of urine. Significant differences in the prevalence and intensity of *S. haematobium* infection were noted between villages. A single dose of PZQ significantly reduced the prevalence of *S. haematobium* infection from 73.2 % to 4.6 % and the geometric mean intensity of infection from 356.1 to 43.3 eggs/10 ml of urine. The cure rates ranged from 89.4 % to 100 %. The egg reduction rates also ranged from 77.6 % to 100 %. Two to three months after the period of transmission, the overall rate of reinfection was 12.6 % and was significantly higher in male children than in female children. The overall prevalence at this period was 13.8 %, which was significantly lower than the prevalence at baseline (73.2 %).

**Conclusion:**

The Niakhar study area remains a hot spot of urinary schistosomiasis in Senegal with differences in transmission between villages. This study suggests that when transmission is strictly seasonal, Praziquantel shows the expected efficacy in reducing the prevalence and intensity of infection, but also a significant effect on the occurrence of reinfection.

## Background

Human schistosomiasis remains a significant public health issue worldwide [1]. Sub-Saharan Africa is the most affected region, encompassing 90 % of the 207 million schistosomiasis cases recorded in the world [1, 2]. The burden of schistosomiasis is exacerbated in low income countries where access to clean water and regular sanitation systems is limited [3, 4]. The disease is caused by six *Schistosoma* species: *S. haematobium*, *mansoni*, *japonicum, intercalatum*, *mekongi and guineensis* [5, 6].

In sub-Saharan Africa, *S. haematobium* and *S. mansoni* are the predominant species causing urogenital and intestinal schistosomiasis, respectively [5, 6]. In areas with permanent water bodies, these two species can be co-endemic [7]. By contrast, *S. haematobium* is only transmitted in seasonal transmission areas, due to the fact that only the snail of the genus *Bulinus*, the intermediate hosts, can resist aestivation during the dry time of year, unlike the snails of genus *Biomphalaria,* the intermediate host of *S. mansoni* [8, 9].

Currently, in endemic areas, the control of schistosomiasis disease is mainly based on chemo-preventative strategies to prevent morbidity later in life due to the chronic infection with *Schistosoma* parasites. School-aged children and communities at high risk of infection are targeted. The treatment consists of a single oral dose of 40 mg/kg Praziquantel (PZQ) after or without a prior diagnosis of *Schistosoma* spp. [3, 10]. PZQ is also the drug of choice for the treatment of schistosomiasis due to its high efficacy against all schistosome species infecting humans [11].

The implementation in the past 10 years of national control programs based on mass PZQ administration in school-age children, has allowed the significant reduction in transmission of schistosomiasis in Sub-Saharan Africa [10]. Despite a few reports of treatment failures [12, 13], the majority of studies showed good efficacy of PZQ, with high egg reduction rates (ERRs) in the urine in foci of intense *S. haematobium* transmission and especially in low-to-moderate transmission areas where the risk of reinfection is generally low [14–16].

The rate and intensity of post-PZQ chemotherapy reinfection varies between schistosome species, the dynamic of the transmission and the level of endemicity [15]. In areas of permanent schistosomiasis transmission, the efficacy of the treatment is enhanced when PZQ is administered when the level of disease transmission is low [17]. In southwestern Niger, the effectiveness of treatment with a single dose of PZQ was better in the village located along a temporary pond than in the village near an irrigated area of the Niger River Valley where the transmission is permanent [18]*.*

In Senegal, *S. haematobium* and *S. mansoni* coexist in 3 regions: the region of the Senegal River Basin (SRB) [19], Kolda [20] and Kedougou [21], respectively in the north, south and south east of the country. The disease is most prevalent along the Senegal River Basin where the prevalence can reach 95 % due to the permanence of transmission, since contact with water is inevitable [7].

In these villages around the SRB and the “lac de Guiers”, several previous studies evaluated the efficacy of one or repeated treatments and monitored the reinfection rates. The results showed a high efficacy of PZQ against *S. haematobium* infections but reinfection occurred rapidly and the prevalence could reach the pre-treatment level [7, 22, 23]. This makes it difficult to control and eliminate the disease in this region despite the many efforts following the outbreak of Richard-Toll [24]. These are the reasons that new approaches to biological control of schistosomiasis are under experimentation in the SRB [25, 26].

By contrast, most parts of the country, except the region of Dakar (the capital), only *S. haematobium* is endemic in the ten other regions and the disease is most prevalent in school-age children [27]. In these regions, the prevalence of urinary schistosomiasis is increasing because of the absence of regular treatments, the opposite of what is happening in the SRB. No information is available on the efficacy of PZQ against *S. haematobium* and reinfection after treatment in these settings where the disease occurs seasonally in temporal ponds and/or backwaters during the rainy season in Senegal. This is the case in the Fatick region, where in 1996, the prevalence ranged from 3 % to 31 % in some villages of the region [27]. In other parts of the region, more recent studies have shown an increased prevalence of up to 39.6 % in 2007 [28] and more than 50 % in 2009 [29].

In the Niakhar area, in central Senegal, the hydro geographical system is only constituted by temporary ponds and backwaters. *S. haematobium* transmission occurs seasonally between July and November with high prevalence and intensity of infection [29]. The population of Niakhar has no access to PZQ and until now, no mass drug administration against *S. haematobium* has been performed. The aims of the present longitudinal study were to assess the efficacy of PZQ-based treatment against *S. haematobium* and to monitor reinfection in Niakhar school-age children.

## Methods

### Study area

**Fig. 1:**
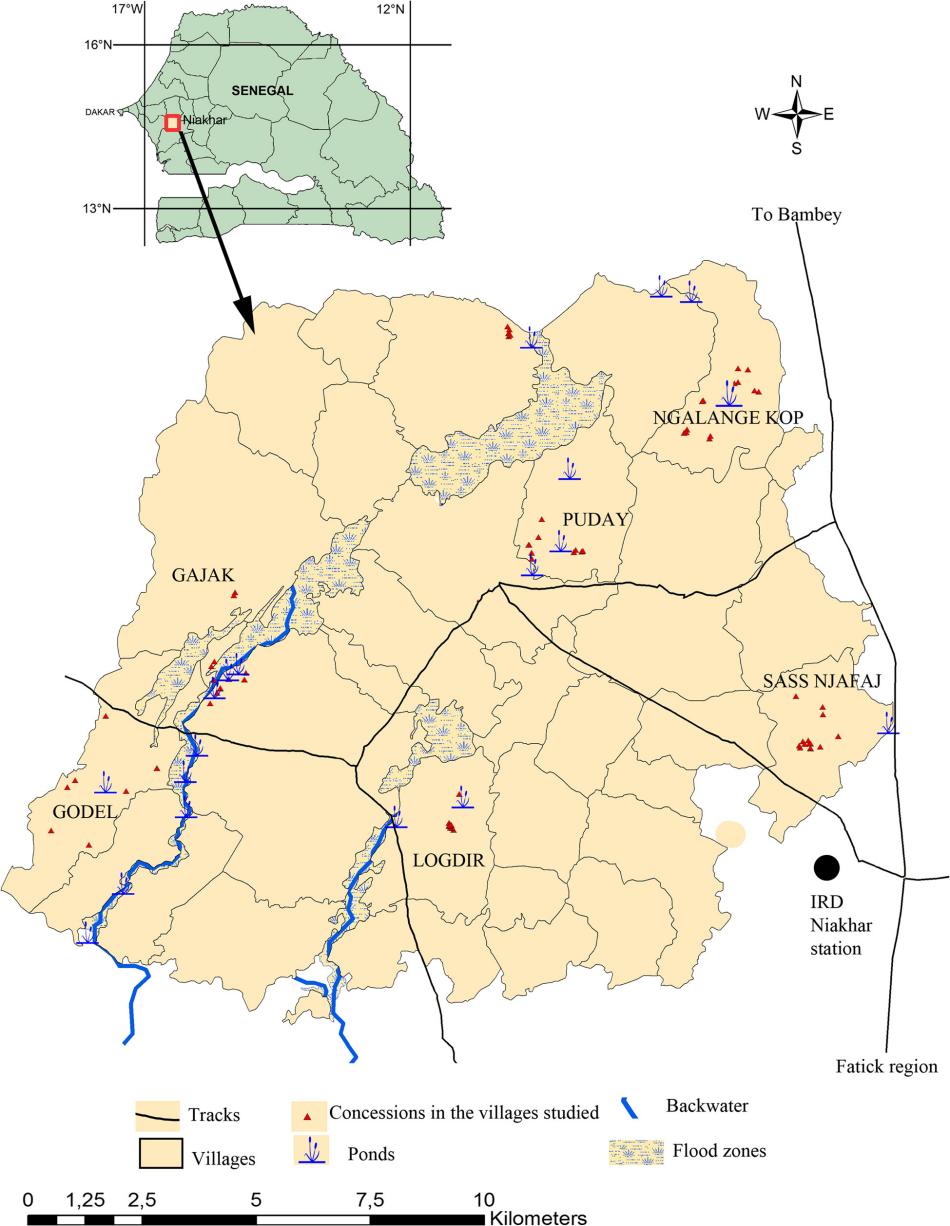
Map of the Niakhar study area showing the water points and the houses in the villages investigated

The study was carried out at Niakhar district (14°30 N, 16°30 W), a demographic survey site located in the region of Fatick (West central Senegal), 135 km east of Dakar, the capital of Senegal, in West Africa. The rainy season spans four months (July through October) [30]. The area is a seasonal transmission focus of urinary schistosomiasis. *S. haematobium* transmission sites consisted of only ponds and backwaters that were made from July to December. In the middle of winter, the area is separated from south to north by the backwater that persists during the dry season. The ponds dry up faster than the backwaters. From January until the end of June, the population of Niakhar is not exposed to *S. haematobium*, as all water collections are dry. The study population included school-age children between 5 and 15 years of age enrolled from six villages of Niakhar: Gajak, Godel, Logdir, Sass njafaj, Ngalagne kop and Puday. In the villages of Gajak and Godel the backwaters are used especially for bathing, swimming, laundry, fetching water, washing pets and fishing while in Sass njafaj, Ngalagne kop, Poudaye and Logdir those activities are done in the ponds, except fishing. Geographical positions of the main water points and the houses selected in each village surveyed were determined using a global positioning system (GPS). The hydrological network varies from village to village. It is very dense at Ngalagne kop, Puday and Logdir where the ponds are near the houses. At Sass njafaj ponds are rare and far from the houses. The same situation is observed at Godel where the populations use one pond, but which is near the houses. At Gajak, there is no pond but the village is bordered by a large backwater near the houses (Fig. 1). There was no mass PZQ administration before the study. A more detailed description of the Niakhar study area has been given elsewhere [29, 31].

**Fig. 2:**
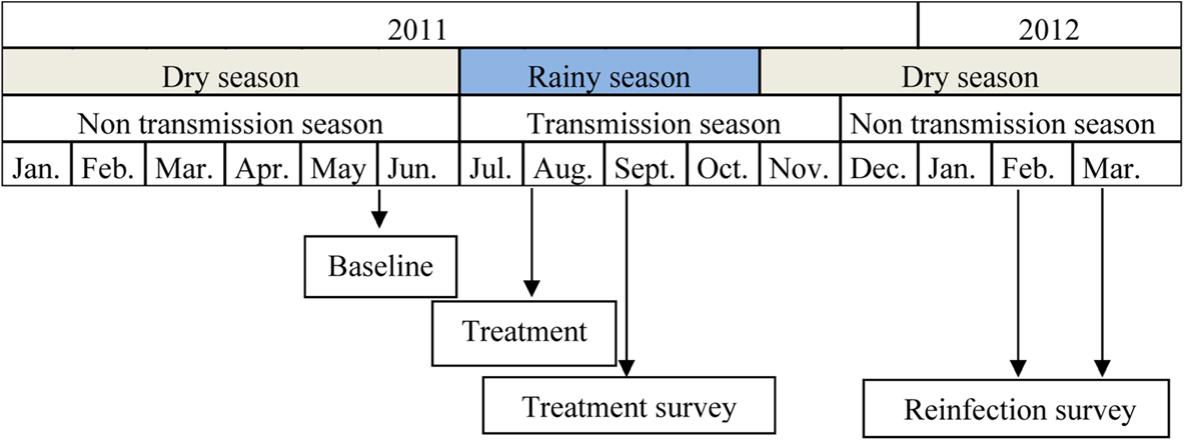
Study design

### Treatment

Praziquantel was given by Merck KGaA; Darmstadt, Germany to the World Health Organization (WHO) for mass distribution by the national program in the fight against schistosomiasis in Senegal. The Niakhar study area is one of the sites selected for five rounds of annual mass treatment against schistosomiasis based on the prevalence of urinary schistosomiasis higher than 50 % [29]. This study took place a year before the beginning of mass treatments in the study zone. The drug was made available to us by the national program and was administered at the standard dose of 40 mg/kg at the health centers of the study zone, and in the presence of a nurse and an agent from the national program.

### Study design

This study is a longitudinal cohort survey before and after treatment with PZQ. The six villages were enrolled based on their *S. haematobium* prevalence reported in a previous study [29]. The study was conducted between June 2011 and March 2012. The inclusion criteria were: i) residence in the studied villages during the rainy season, ii) an age of between 5 and 15 years and iii) consent to participate in the study. At the beginning of the study, in June 2011, the population of study was not in contact with water collections because the Niakhar area was dry from December 2010 to June 2011. The study was conducted in three successive phases with the same children surveyed at each point of time (Fig. 2):

- Study of the baseline prevalence of *S. haematobium* in Niakhar: this phase was conducted in June 2011, before the beginning of the 2011 transmission season. At these points in time, a baseline urine examination was conducted in all children selected for the study. A child is considered infected by *S. haematobium* if at least one egg is found in a urine sample during microscopic observation.
- Monitoring the efficacy of PZQ treatment: in early August 2011, after the baseline phase, PZQ (40 mg/kg) was administered to all children selected, whether infected or not. The treatment was done in the middle of the transmission season because PZQ was not available between June and July. Five weeks after treatment, in September 2011, one urine sample was collected from each child to assess the efficacy of the treatment. Any individual was considered cured if no egg was found in the urine sample examined.
- Monitoring reinfection: reinfection was monitored from February to March 2012 when all water collections are totally dry and *S. haematobium* transmission does not occur. Urine samples were collected to determine the rate of reinfection. This monitoring of reinfection involved only children who were positive and subsequently became negative and then positive again. Each negative individual during the September 2011 survey of PZQ treatment efficacy was considered reinfected if at least one egg was found in the urine sample.

### Urine Sample collection and microscopic analysis for the detection of S. haematobium eggs

Urine samples were collected door-to-door between 10:00 am and 2 p.m., and transferred to the Niakhar laboratory for parasitological analysis during the same day. The urine sample was shaken to ensure the dispersal of eggs and then ten milliliters were taken and filtered through a Millipore® SX0001300 Swinnex® Syringe Filter. Filtration was followed by microscopic examination for detection of *S. haematobium* eggs according to the method developed by Plouvier *et al*. [32]. The infection intensity was classified as light (1–49 eggs/10 ml of urine) or heavy (≥50 eggs/10 ml of urine), as defined by the World Health Organization [33].

### Ethical approval

The study was part of a larger investigation on schistosomiasis epidemiology, transmission and control in Senegal and which was approved by the Senegalese National Ethics Committee (reference No.SN11/57). The objectives of the study were explained to the children and to their parents or legal guardians, from whom written informed consent was obtained.

### Statistical analysis

Data from each village were recorded using Epi-Info, version 3.5.1 and analyzed using STATA 11.1. The relationships between characteristics of *S. haematobium* infection (prevalence, intensity and reinfection) and other variables, such as the location of villages and the sex and age of children, were tested at baseline. Prevalence comparisons were performed using the chi-squared test and Fisher’s exact modification of the 2 × 2chi-squared test [34]. For infection intensity values, the Geometric Means of Williams was used to calculate the Geometric Eggs Mean Count (GEMC) in only positive individuals. PZQ efficacy was measured by determining both cure rate (CR) and egg reduction rate (ERR). The CR is the percentage of children positive for egg-patent infection becoming negative after treatment. The ERR is the percentage reduction in GEMC, as measured by *S. haematobium* eggs, after drug treatment of children with egg-patent infections at baseline. The ERR was calculated as [1 − (GEMC after treatment/ GEMC before treatment)] × 100 [35]. GEMC among different groups was compared using ANOVA. In all case, a *P*- value < 0.05 was taken to indicate statistical significances.

## Results

### Baseline prevalence and intensity of S. haematobium infection by village, sex and age

Six villages Gajak, Godel, Logdir, Ngalagne, Puday and Sass njafaj were selected for the study. A total of 329 subjects were enrolled at baseline. The average age and the sex ratio m/f were 8.8 (SD, 3.1) years and 1.4, respectively. The population was arbitrarily classified into three age groups: 5 to 7, 8 to 10 and 11 to 15 years. Table 1 summarizes the demographic characteristics of the cohort.

**Table 1:** Demographic characteristics of the studied population

Villages	Sample size at baseline	Age mean (SD^a^) in years	Sex ratio (M/ F)
Gajak	98	7.8 (2.6)	0.8
Godel	52	8.2 (2.8)	0.8
Logdir	31	8.5 (3.2)	0.6
Ngalagne kop	52	9.1 (2.8)	1.5
Puday	52	8.3 (3)	1.5
Sass ndiafaj	44	9.5 (2.7)	1.3
Total	329	8.8 (3.1)	1.4

^a^Standard Deviation

Among the 329 individuals examined at baseline, 241 (73.2 %) were infected by *S. haematobium* and the other 88 were negative (Table 2). The prevalence of *S. haematobium* infection was 92.3 %, 80.5 %, 78.8 %, 71.4 %, 61.5 % and 50 % at Ngalagne kop, Logdir, Gajak, Poudaye, Godel and Sass njafaj, respectively. The GEMC varied from 1573.9 eggs/10 ml of urine in the village of Ngalagne kop to 128.3 eggs/10 ml of urine in Logdir. Significant differences in the prevalence and intensity of infection were noted between villages (*p* < 0.001). The infection rate was higher in males (80.2 %) than females (64.1 %) (*p* < 0.05). The same trend was observed with the GEMC, which was 544.1 and 177.0 eggs/10 ml of urine for males and females, respectively. Significant differences in prevalence and GEMC according to sex were noted (*p* < 0.05) (Table 3). Prevalence increased according to age with 62.4 %, 80.9 % and 81.9 % in the 5 to 7, 8 to 10 and 11 to 15 age groups, respectively. But the GEMC was higher in the 8 to 10 year age group. Significant differences in the prevalence and intensity of infection were noted between the 5 to 7 year age group and the others (*p* < 0.001) (Table 3).

**Table 2:** The prevalence and intensity of *S. haematobium* infections in children from six villages in the Niakhar study area in Central Senegal before treatment with PZQ, 5 weeks post treatment and reinfection levels at 2 to 3 months after draining of ponds and backwater

Characteristics *S. haematobium* infection								
Villages	Gajak	Godel	Logdir	Puday	Ngalagne kop	Sass njafaj	Total	P-value
Baseline (June 2011)								
No. of children infected/examined	77/98	32/52	25/31	37/52	48/52	22/44	241/329	<0.001
Prevalence (95 % CI)	78.6 % (67.2-83.8)	61.5 % (47.9-73.5)	80.6 % (63.7-90.8)	71.1 % (57.7-81.6)	92.3 % (81.8-96.9)	50 % (35.8-64.2)	73.2 % (68.2-77.2)	
Numbers of infected children treated with PZQ	75	32	25	36	47	22	237	
GM egg count/10 ml of urine (95 % CI)	169.8 (119.2-241.8)	189.3 (105.6-339.4)	128.3 (73.8-222.8)	966.6 (581.2-1607.5)	1573.9 (1208.2-2050.4)	276.8 (132.2-580.1)	356.1 (286.1-443.3)	<0.001
Treatment (August 2011)								
Follow-up efficacy of PZQ 5 weeks post treatment (in September 2011)								
No. of children infected/examined	7/96	1/52	0/31	1/51	5/51	1/44	15/325	
Prevalence (95 % CI)	7.3 % (3.6-14.3)	2 % (0.3-10.3)	0 % (0–11)	1.9 % (0.3-10.1)	9.8 % (4.2-20.6)	2.3 % (0.4-11.8)	4.6 % (2.7-7.4)	0.219
Numbers of infected treated examined	75	32	25	36	47	22	237	
No of children cured (CR in %)	68 (90.6 %)	31 (96.8 %)	25 (100 %)	35 (97.2 %)	42 (89.4 %)	21 (95.4 %)	222 (93.7 %)	0.7
GM egg count/10 ml of urine (95 % CI)	38.1 (9.1-159)	30.0	.	35.0	64.9 (25.2-167.6)	25.0	43.3 (23.1-81.2)	0.144
ERR in %	77.6 %	84.1 %	100 %	96.4 %	95.9 %	90.9 %	87.8 %	0.92
Reinfection after treatment (September to November 2011)								
Follow-up reinfection 2 to 3 months after transmission period in February and March 2012								
No. of children infected/examined	15/96	2/52	2/31	14/51	11/51	1/44	45/325	
Prevalence (95 % CI)	15.6 % (9.7-24.2)	3.8 % (1.1-12.9)	6.4 % (1.8-20.2)	26.9 % (16.7-40.2)	21.7.% (12.5-34.6)	2.3 % (0.4-11.8)	13.8 % (10.5-18)	0.002
GM egg count/10 ml of urine (95 % CI)	99.9 (45.1-221.3)	215.6 (0–1.1e + 13)	462.5 (0–5.7e + 11)	30.7 (10–94.5)	12.0 (4.4-33.1)	506.0	48.1 (26.6-86.8)	0.01
No. of children re-infected/surveyed	8/89	1/51	2/31	13/50	6/46	0/43	30/310	
Reinfection rate (95 % CI)	8.9 % (4.6-16.7)	1.9 % (0.3-10.3)	6.4 % (1.7-20.7)	26 % (15.8-39.5)	12.8 % (5.9-25.2)	0 % (0.0-10)	9.7 % (6.9-13.5)	<0.001

**Table 3:** The prevalence and intensity of *Schistosoma haematobium* infections in children from six villages in the Niakhar study area in Central Senegal at baseline, 5 weeks post treatment and reinfection levels at 2 to 3 months after draining of ponds and backwater, relative to gender and age

Characteristics *S. haematobium* infection							
		Gender			Age		
Variables	Female	Male	P-value	5 - 7 years	8 - 10 years	11 - 15 years	P-value
Baseline (June 2011)							
No. of children infected/examined	91/142	150/187		88/141	85/105	68/83	
Prevalence (95 % CI)	64.1 % (55.9-71.5)	80.2 % (73.9-85.3)	0.001	62.4 % (54.2-69.9)	80.9 % (72.4-87.3)	81.9 % (62.3-88.7)	0.001
Numbers of infected subject treated with PZQ	91	146		88	83	66	
GM egg count/10 ml of urine (95 % CI)	177.0 (123.4-253.8)	544.1 (421.4-702.6)	0.001	238.9 (164.4-347.3)	455.6 (313–662.9)	438.7 (299.1-643.3)	<0.001
Treatment (August 2011)							
Follow-up efficacy of PZQ 5 weeks post treatment (in September 2011)							
No. of children infected/examined	3/142	12/183		6/141	8/103	1/81	
Prevalence (95 % CI)	2.1 % (0.7-6)	6.5 % (3.7-10.9)	0.080	4.2 % (1.9-8.9)	7.7 % (3.9-14.4)	1.2 % (0.2-6.7)	0.200
No. of infected treated examined	91	146		88	83	66	
No of children cured (CR in %)	88 (96.7 %)	134 (91.8 %)	0.7	82 (93.2 %)	76 (91.7 %)	64 (96.9 %)	0.5
GM egg count/10 ml of urine (95 % CI)	15.7 (3.7-65.4)	55.8 (25.5-112.9)	0.06	26.5 (8–87.7)	67.0 (26.9-166.5)	25.0	0.109
ERR in %	91.1 %	89.7 %	0.99	88.9 %	85.3 %	94.3 %	0.99
Reinfection after treatment (September to November 2011)							
Follow-up reinfection 2 to 3 months after transmission period in February and March 2012							
No. of children infected/examined	11/142	34/183		22/141	14/103	9//81	
Prevalence (95 % CI)	7.7 % (4.3-13.3)	18.6 % (13.1-24.8)	0.010	15.6 % (10.5-22.5)	13.6 % (8.2-21.5)	11.1 % (5.9-19.8)	0.486
GM egg count/10 ml of urine (95 % CI)	37.4 (37.4-12.38)	52.4 (25.2-109.2)	0.012	73.1 (31.8-168.3)	27.8 (9.4-82.4)	38.6 (5.6-263.7)	0.307
No. Of children re-infected/surveyed	8/139	22/173		16/135	8/96	6/81	
Reinfection rate (95 % CI)	5.7 % (2.9-10.9)	12.7 % (8.5-18.5)	0.001	11.8 % (7.4-18.4)	8.3 % (4.3-15.6)	7.4 % (3.4-15.2)	0.400

### Efficacy of PZQ against S. haematobium according to village, sex and age

Among the 241 infected children, 237 were treated and monitored for PZQ efficacy. Four children were not treated due to their absence and are excluded in the study but they were referred to health centers where the rest of PZQ was left. A total of 222 children were egg negative after treatment. The overall CR and ERRs were 93.7 % and 87.8 %, respectively. After treatment, the overall prevalence and the GEMC were significantly reduced. The prevalence of *S. haematobium* infection decreased from 73.2 % to 4.6 % (*p* < 0.05). Significant decreases in prevalence were noted in each village (*p* < 0.05). Prevalence after PZQ treatment was 9.8 %, 7.3 %, 2.3 %, 2 %, 1.9 % and 0 %, in the villages of Ngalagne kop, Gajak, Sass njafaj, Godel, Puday and Logdir, respectively. The same trend was observed with the intensity of infection. The GEMC decreased from 356.1 to 43.3 eggs/10 ml of urine (*p* < 0.05). CRs were high in all six villages and varied from 89.4 % at Ngalagne kop to 100 % at Logdir. The ERRs were also high and ranged from 77.6 % to 100 % in these same villages. No significant difference was observed in the CR and ERRs between villages (p > 0.05) (Table 2).

In the 237 positive children treated, 222 were totally cured and eggs were found in urine samples of only 15 (6.3 %) children. Among these 15 children at baseline, five had excreted over 49 eggs/10 ml of urine and three of them lived in the village of Ngalagne kop where the highest GEMC (1 573.9 eggs/10 ml of urine) was recorded during this study.

The efficacy of PZQ against *S. haematobium* infection based on age is shown in Table 3. After treatment, a significant reduction in the prevalence was observed. In each age group, pre-treatment prevalence was significantly reduced from 62.4 % to 4.2 %, 80.9 % to 7.7 % and 81.9 % to 1.2 % in the 5 to 7, 8 to 10 and 11 to 15 age groups, respectively (*p* < 0.05). The highest CR (96.9 %) and ERR (94.3 %) were recorded in the 11 to 15 age group. These parameters were lowest in the 8 to 10 year age group and were at 91.7 % and 85.3 % for the CR and ERRs, respectively. But there was no significant difference in the CR between age groups (*p* < 0.05). The results broken out by sex are also available in Table 3.

### Reinfection by villages, sex and age

The study of reinfection involved the 222 children who were cured after PZQ administration. Among these children who were negative after control of the treatment in September 2011, 28 (12.6 %) reinfection cases from the 2011 transmission season were detected from February to March 2012. The overall prevalence in the population at this point in time was 13.8 (Table 2). No reinfection occurred in the village of Sass where the lowest baseline prevalence was recorded. In the other villages, reinfection rates were 37.1 %, 11.9 %, 10.3 %, 8 % and 3.2 %, at Puday, Ngalagne kop, Gajak, Logdir and Godel, respectively. The patterns of reinfection with *S. haematobium* showed statistical difference between villages (*p* < 0.001). Compared to the baseline, in each village, the prevalence was significantly lower after the period of reinfection (*p* < 0.05). The overall GEMC (48.3 eggs/10 ml of urine) was significantly lower than the baseline (356.1 eggs/10 ml) (*p* < 0.001) (Table 2). The reinfection rate was significantly higher in males (12.7 %) than in females (5.7 %) (*p* < 0.05). The reinfection rate was more significant in children aged 5 to 7 years (17.1 %) than in the groups of 8 to 10 and 11 to 15 which had quite quantative statement reinfection rates: 10.5 % and 9.4 %, respectively. However, there was no significant difference in *S. haematobium* reinfection between the different age groups (p > 0.05) (Table 3).

## Discussion

In the present study, high prevalence and GEMC of *S. haematobium* infection are observed at baseline in the area of Niakhar. These results are in line with a recent investigation that has shown high prevalence and intensity of *S. haematobium* infection in the Niakhar district [29]. This high level of infection also confirms that Niakhar is a hot spot for *S. haematobium* transmission in Senegal. In endemic areas, the level of *S. haematobium* transmission is very significant in school-age children between 5 and 15 years of age [36]; this may explain the high prevalence noted in this study, which targeted only school-age children. This epidemiological situation in Niakhar, marked by a high level of urinary schistosomiasis transmission is also attributable to the lack of health education, poor sanitation and also the lack of safe water resulting in permanent contact with ponds and backwaters which are the main areas of *S. haematobium* transmission during the rainy season. In addition, before this investigation, no strategy, i.e. PZQ mass administration, has been implemented to control the transmission of the disease [14].

In this study, a high prevalence of *S. haematobium* was noted in all the study areas; however, the level of infection was heterogeneous between villages. The distribution of ponds and backwaters may result in heterogeneity in the prevalence and intensity of *S. haematobium* infections between study sites. This may have implications for the heterogeneity found in the transmission of the disease in Niakhar, where the highest level of *S. haematobium* infection was recorded in villages with the highest density of ponds and backwaters which are near the households like at Ngalagne kop, Logdir, Gajak, Puday. The lowest level of transmission is found in Sass njafaj, which has a poor pond system with limited frequentation by the population. Therefore, the results of this study indicate once again the importance of the hydrogeographical network in the epidemiology and transmission of *S. haematobium* [37, 38]. This heterogeneity between villages may also be explained by access to tap water present in some households in Niakhar, or by social behavior [29]. In the Niakhar study area, it has been shown that the level of frequentation of ponds and backwaters is strongly linked to social behavior and that contact with these water collections is more significant in male children, particularly those between 8 and 12 years of age [29]. Therefore, this could explain the difference in infection observed in male children compared to females and the highest prevalence in boys between the 8 to 10 and 11 to 15 years age groups. This situation in Niakhar required implementation PZQ mass administration targeting each year children aged 5 to 15 years and also health education to reduce contact with infested water.

Until now, the efficacy of control strategies based on PZQ mass administration is poorly documented in seasonally *S. haematobium* transmission foci in Senegal. This study revealed that a single dose of 40 mg/kg of PZQ significantly reduced the infection prevalence and intensity in all villages of the study area, confirming the efficacy of PZQ in the treatment of urinary schistosomiasis. The CR and ERRs obtained are higher than those observed in previous studies conducted in seasonal *S. haematobium* transmission foci [37] despite that the fact that the treatment was done during the transmission period due to the none accessibility of PZQ after baseline survey in June and July.

Indeed, to better assess the impact of treatment, PZQ should be administered during the low or non-transmission period. However, this does not affect the treatment outcome, since in Niakhar the transmission of *S. haematobium* is strictly seasonal and occurs from July to November; hence the adult worms from the previous transmission periods were exposed to the August 2011 PZQ treatment. However, the collection of one urine sample per child due to limited resources could be a limit explaining the high CR obtained in this Niakhar study. The examination of two or more urine samples per child on different days and more than filtration should be more appropriate for estimating the CR and ERR because of the day-to-day variation in egg excretion in urine [39].

Five weeks after treatment, eggs were found in 6.3 % of treated children in this study, particularly in the boys group, where the highest pre-treatment GEMC was observed. The same trend was also observed in young Gambians [37]. In this Niakhar study, for each child, the PZQ was given under the supervision of a nurse at the health center; therefore, the probability that these excreted eggs come from non-compliance with PZQ is unlikely. However, egg excretion is very common after treatment with PZQ [15] and *S. haematobium* eggs that have been dead for some time can be excreted some months after the drug administration [40]. Therefore, to fully assess PZQ efficacy, it would more appropriate to determine by hatching the viability of the eggs still being excreted by some individuals.

The treatment period could also be the cause of the presence of eggs in these children. Indeed, an infection could occur between July and August 2011 just before PZQ administration and when the rainfall and the frequentation of transmission sites are high. The PZQ administered during this period is not effective against the immature stages of the parasite infecting the children [41]. These immature forms could reach the adult stage and their eggs can be detected during evaluation of the efficacy of PZQ in September 2011. Therefore, this rate of egg excretion after treatment could be very low or even zero if the treatment was administered during non-transmission period between March and May and the control carried out before July, in addition to the egg viability assessment.

The present study nevertheless shows the difficulty with a single dose of PZQ to cure totally some children in whom the intensity of the infection was very high before treatment [42]. A supplementary dose of PZQ could be necessary to completely cure this category of infected individuals in the Niakhar study area. However, in this strictly seasonal transmission area there was no difference in the CR and ERRs of *S. haematobium* between villages after PZQ treatment. It seems that these parameters were not linked to the intensity of infection in the villages at baseline before administering PZQ. These results are contrary to those observed in highly endemic villages in Côte d’Ivoire where transmission is permanent [43] and indicate a clear association between PZQ efficacy and infection intensity prior to treatment for *S. mansoni* [44].

The use of PZQ always remains a valuable strategy for morbidity prevention and the control of *S. haematobium* transmission. Indeed, prevalence and infection intensity after reinfection decreased significantly compared to the baseline because a low rate of reinfection was observed at the end of the 2011 transmission season in the study village. This low level of reinfection could be explained by a low level of transmission in the study site but also by the fact PZQ treatment considerably reduced children harboring eggs, thus resulting in a decrease of the snails infection [45]. Another factor might be the ecology of the area, which is characterized by a short transmission period and that some children may have the chance of being infected in an interval of one or even two years. Few infections were reported in an area where transmission sites were composed of seasonal rain-fed ponds when PZQ treatment was administered and monitored in the non-transmission period [37]. This is also the case in some transmission foci where populations also use only temporal ponds [18, 43]. These conditions are contrary to what happens in the SRB, where transmission and reinfection of *S. haematobium* and *S. mansoni* occurs throughout the year and prevalence after six-month period following treatment can reach the pre-treatment levels, especially for *S. mansoni* [7, 23]. During this study, reinfection is more significant in males than in females. This difference may be due to cultural, behavioral and social factors that put boys in more contact with water collections than girls [29].

The current results show that the efficacy of PZQ against *S. haematobium* was satisfactory and that treatment has a considerable impact on the morbidity due to eggs and affect the occurrence of reinfection in children surveyed in all the villages studied. This suggests that PZQ mass administration between March and May could be used to significantly reduce child carriers of *S. haematobium* and to control the disease in order to move towards elimination of urinary schistosomiasis in seasonal foci in Senegal.

## Conclusion

The baseline results confirm that urinary schistosomiasis is a public health problem in the Niakhar District. A single dose of 40 mg/kg of PZQ has a significant impact on the prevalence and the intensity of infection. This paper suggest that when transmission is not constant and large-scale as it was in the Senegal River Basin, PZQ shows the expected efficacy and a significant effect on the occurrence of reinfection due to the strict seasonality of transmission. These results provide a basis for possible implementation of a urinary schistosomiasis control program in the district of Niakhar but also in other foci were transmission is only seasonal in Senegal.
